# NeuroControl of movement: system identification approach for clinical benefit

**DOI:** 10.3389/fnint.2015.00048

**Published:** 2015-09-08

**Authors:** Carel G. M. Meskers, Jurriaan H. de Groot, Erwin de Vlugt, Alfred C. Schouten

**Affiliations:** ^1^Department of Rehabilitation Medicine, VU University Medical CenterAmsterdam, Netherlands; ^2^Department of Rehabilitation Medicine, Leiden University Medical CenterLeiden, Netherlands; ^3^Department of Biomechanical Engineering, Delft University of TechnologyDelft, Netherlands; ^4^Laboratory of Biomechanical Engineering, Institute for Biomedical Technology and Technical Medicine (MIRA), University of TwenteEnschede, Netherlands

**Keywords:** afferent feedback modulation, neuromechanics, system identification, ageing, stroke, movement disorders

## Abstract

Progress in diagnosis and treatment of movement disorders after neurological diseases like stroke, cerebral palsy (CP), dystonia and at old age requires understanding of the altered capacity to adequately respond to physical obstacles in the environment. With posture and movement disorders, the control of muscles is hampered, resulting in aberrant force generation and improper impedance regulation. Understanding of this improper regulation not only requires the understanding of the role of the neural controller, but also attention for: (1) the interaction between the neural controller and the “plant”, comprising the biomechanical properties of the musculaskeletal system including the viscoelastic properties of the contractile (muscle) and non-contractile (connective) tissues: neuromechanics; and (2) the closed loop nature of neural controller and biomechanical system in which cause and effect interact and are hence difficult to separate. Properties of the neural controller and the biomechanical system need to be addressed synchronously by the combination of haptic robotics, (closed loop) system identification (SI), and neuro-mechanical modeling. In this paper, we argue that assessment of neuromechanics in response to well defined environmental conditions and tasks may provide for key parameters to understand posture and movement disorders in neurological diseases and for biomarkers to increase accuracy of prediction models for functional outcome and effects of intervention.

## Introduction

Posture and movement disorders in neurological diseases like stroke and in ageing are of increasing clinical concern; due to both an increasing incidence and prevalence as a result of aging of the society as well as increasing awareness of socioeconomic impact, i.e., disability and as a result, loss of autonomy. Disability can be translated to the inability to adequately cope with daily environmental challenges.

**Figure 1:**
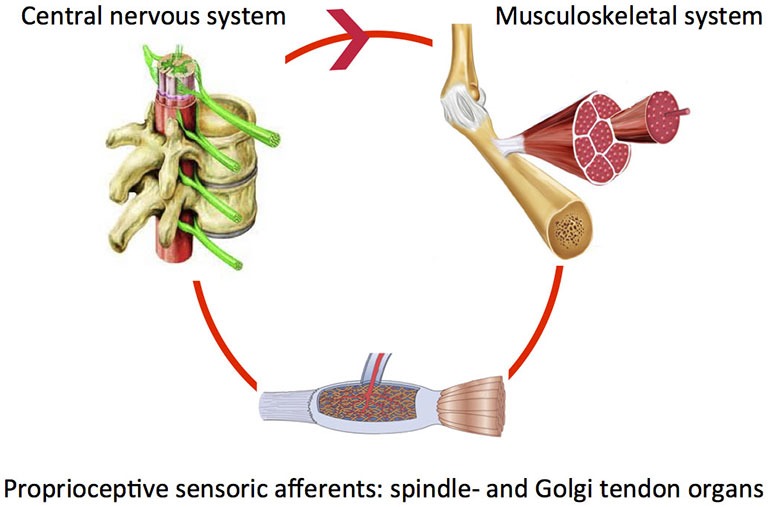
**Closed loop of sensing, processing and appropriate action: peripheral (spinia) reflexes**.

Our body segments interact with fixed and moving obstacles and objects in the environment. This involves exchange of mass, energy, linear or angular momentum in order to produce adequate posture and movement patterns. For example when reaching and grasping objects, the right amount of muscle force is required to properly control the joint impedance. During walking, the mechanical interaction between the leg segments and other body parts requires continuous control. To reduce the impact of posture and movement disorders in neurological diseases it is crucial to investigate how the “altered” system adapts to varying tasks and environmental conditions. Both the neural system (controller) and the muscles (“motor”) are end-effectors at the level of the joint. System adaptability may subsequently be translated to the modulatory capacity of the neuromuscular system. Understanding of the modulatory capacity of the neuromuscular system in terms of mechanics, i.e., neuromechanics will ultimately allow for relating specific system states to the global level of function. Of key importance is the notion that components determining the neuromechanics continuously interact within a closed loop. For example, the proprioceptive muscle spindle and Golgi tendon organs sense muscle states, information is processed and subsequently fed back to the muscle (basic control loop, Figure 1).

In this paper we argue that modulatory capacity of the neuromechanical system can best be assessed when properties of the neural and biomechanical system are addressed synchronously. This paper illustrates the role for neuromechanics in understanding and eventually addressing movement disorders in a variety of diseases and clinical states. System identification (SI) is the required technique to address the closed-loop interaction between neural controller and biomechanical, i.e., musculoskeletal system. Neuromechanical parameters in response to environmental challenges may provide: (1) for key parameters to understand posture and movement disorders in neurological diseases; (2) may be used as biomarkers to increase accuracy of prediction models for functional outcome and; (3) evaluate the effects of intervention.

## The Clinical Problem

### Movement Disorders in Neurological Diseases

Loss of mobility after upper motor neuron diseases like stroke is still conceptually being related to the phenomenon of spasticity, i.e., muscle hypertonia with velocity dependent resistance of joint to passive stretching (Lance, 1980). However, spasticity or signs of exaggerated reflex activity are typically assessed under passive conditions. Hence it may not be surprizing that the relation between degree of spasticity at the level of joints and global functional level is not straightforward (Ada et al., 1998). It has become evident that upper motor neuron diseases like stroke and cerebral palsy (CP) also result in secondary biomechanical changes of muscles, tendons and connective tissue (Vattanasilp et al., 2000; Hof, 2001; Lieber et al., 2004; Gracies, 2005a,b; Dietz and Sinkjaer, 2007; Mirbagheri et al., 2009; Smith et al., 2011). Moreover, it is increasingly well agreed that mobility disorders are the result of a complex interplay between primary neural factors and secondary biomechanical changes which is environment- and task dependent (van der Krogt et al., 2012) and may change over time, e.g., during the recovery phase after stroke (van der Krogt et al., 2015) and/or due to chronological ageing (Groenewegen et al., 2012).

Proper planning of interventions to improve mobility requires understanding of aforementioned interplay at any given moment in time. This requires identification of neuromechanical factors that contribute to improper limb impedance, i.e., resistance to external manipulation under various environmental and task dependent conditions (Krutky et al., 2010; van der Krogt et al., 2012). Observed structural changes, i.e., shortening and stiffening of muscles with loss of sarcomeres, stiffening of muscles through fibrosis and changing tendon properties may be concomitant detrimental outcome secondary to impaired neural control or may compensate for the primary detrimental effects of impaired neural control. On which component should interventions be aimed and at what stage? What is the limiting factor during functional tasks? Increased stiffness around limbs in upper motor neuron disease is commonly treated by either attempts to lower reflex activity or stretching of viscoelastic tissue by e.g., botulinum toxin, splinting, casting or surgery, depending on the assumed main contributor to the observed limb dynamics. Targeted intervention requires identification of neural and non-neural, biomechanical components, which cannot be separated by task (instruction) alone, i.e., as under both passive vs. active conditions neural and non-neural contributors play a role, although their relative contributions may differ. Also, appearance of neural and non-neural contributors both depend on manipulations of acceleration, speed and position.

Evidence is emerging that in stroke patients, rehabilitation induces compensatory strategies, instead of addressing primary neurological repair, i.e., restitution (Kwakkel et al., 1999, 2006; de Haart et al., 2004; Geurts et al., 2005; Buurke et al., 2008; van Kordelaar et al., 2013). Stroke patients unable to fully extend shoulder and elbow because of a flexion synergy, appeared to solve a reaching task problem by leaning forward i.e., exhibiting compensating trunk movements. Similarly, from the absence of changes in timing of muscle activity patterns, it was concluded that functional gait improvement in stroke patients might be more related to compensatory strategies than restitution of muscle coordination patterns in the affected leg (Buurke et al., 2008). Asymmetry in weight bearing in stroke patients decreased during rehabilitation after stroke but increased again under demanding circumstances (de Haart et al., 2004). Thus, observed improvement in a patient’s capacity to deal with environmental challenges is most likely due the compensatory part. Moreover, interventions to improve functionality in patients after stroke with robotic therapy (Klamroth-Marganska et al., 2014) or early- applied constraint induced movement therapy (Kwakkel et al., Accepted) do not seem to address true neurological repair (Kwakkel and Meskers, 2014). Next to lack of fundamental understanding of functional recovery, i.e., adaptability to environmental challenges, assessment tools are lacking to properly identify restitution from compensation with high resolution. Neuromechanics may fill the void.

### Mobility/Balance Impairments and Falls in the Elderly

Multiple integrated systems are involved in balance, which all are prone to age related deterioration (Sturnieks et al., 2008; Engelhart et al., 2014; Pasma et al., 2014a), i.e., proprioception, vision and vestibular function. Age related impairments of the motor system are mainly characterized by sarcopenia, i.e., loss of muscle mass: an important clinical problem in elderly (Rolland et al., 2008; Bijlsma et al., 2013). Sarcopenia implies a reduction in parallel sarcomeres affecting muscle strength. However, ageing is also related to loss of sarcomeres in series (Narici et al., 2003), increased tendon compliance (Narici et al., 2008), architectural changes, i.e., a decrease in fiber pennation angle (Narici et al., 2003), and selective atrophy of muscle IIa fibers (Brown and Hasser, 1996). Force-length and force- velocity relations may become sub-optimal (Narici et al., 2008; Raj et al., 2010). Muscle power is generally more affected than muscle force, which in turn is more affected than muscle mass (Macaluso and De Vito, 2004). Changes in fiber type composition and architecture may be responsible next to the changes in neural factors, a reduction in motor unit number and thereby change in recruitment (Evans, 1997) and changes in the neuromuscular junction (Rudolf et al., 2014). A tight interaction between sarcopenia and changes of the neural controller has been suggested (Kwan, 2013). Cognitive capacity is also suggested to play a role in balance (Maki and McIlroy, 2007). Low cognitive status, i.e., defined with respect to normal cognition based on cut-off values of the Mini-Mental State Examination, Montreal Cognitive Assessment and Visual Association Test, was found to be associated with a lower ability to maintain balance in elderly outpatients (Stijntjes et al., 2014). Longitudinally, impairment in cognition was suggested to precede loss of muscle strength in the oldest old (Taekema et al., 2012). Primary deficits may be compensated for by secondary adaptive strategies, e.g., co-contraction to increase stability (Milner, 2002; Benjuya et al., 2004). Unreliable sensory input may be actively down weighted in favor of reliable information in a redundant system: a process called sensory reweighting (Peterka, 2002; Pasma et al., 2012; Assländer and Peterka, 2014). In elderly, motor function has been found to be associated with an increased cognitive demand (Ranking et al., 2000); however this compensation may be detrimental in case of double tasking (Schaefer and Schumacher, 2011) or cognitive decline (Stijntjes et al., 2014).

Quantification of interrelations of the age-related factors and identifying adaptive strategies is essential for understanding and designing proper intervention in case of mobility/balance impairments and falls: which system to address by training, stimulation or pharmacological intervention; sensory, sensorimotor integration (coordination); cognition (double tasking) or the motor part (strength and power training). Neuromechanical analysis is a promising method to observe these interrelated properties.

## The Nervous System: Afferent Feedback Modulation and Supraspinal Control

**Figure 2:**
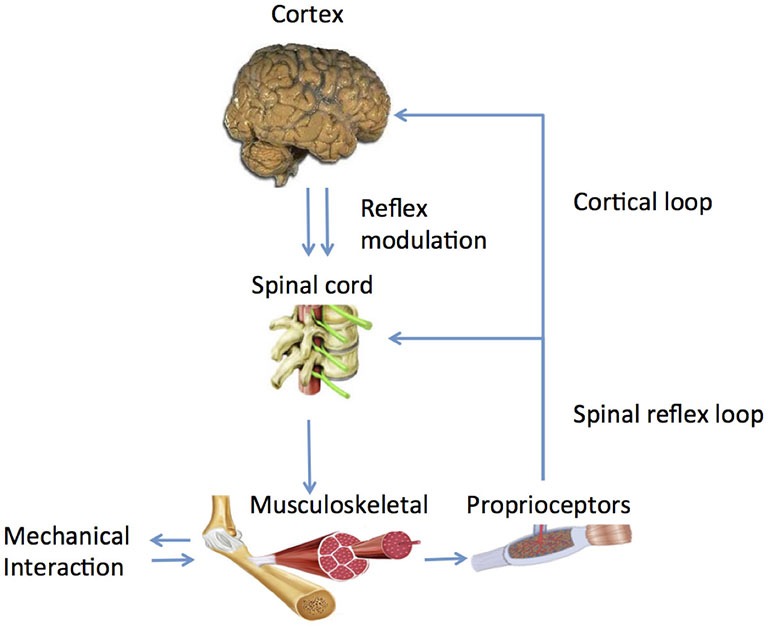
**Main human control loops consisting of a peripheral, spinal loop and a central, cortical loop**. Reflex modulation takes its effect in the peripheral loop while it is mediated by suprapspinal centers and peripheral afferent signals.

Human afferent feedback loops can be discerned into a spinal and a supraspinal loop (Figure 2). Key questions are: (1) what is the nature of altered properties of (supraspinal) control and in what way do they relate to the observed movement disorders? and (2) What are underlying neurophysiological processes and where are they located?

Properties of the neural controller were studied extensively by experiments evoking reflex responses by electrical stimulation (Lourenço et al., 2006) or mechanical perturbation (Lee and Tatton, 1982; Lewis et al., 2005; Pruszynski et al., 2011) exhibiting short and long latency reflex responses. The fact that the long latency reflex appears to be depressed by the known group II afferent blocker Tizanidine (Grey et al., 2001; Maupas et al., 2004; Meskers et al., 2010) is regarded supporting evidence for the at least partial mediation of the long latency reflex by group II afferents and the involvement of group II pathways in upper and lower limb spasticity. A number of underlying pathophysiological processes have been described, i.e., persistent inward currents, diminished post-activation depression and loss of presynaptic inhibition that induces hyper excitability of motor neurons and afferents, probably as a compensation for reduced functional neural and muscular activation in neural disorders (D’Amico et al., 2014). The concept of hyperreflexia translates to the clinical picture of enhanced reaction to tendon taps and velocity dependent resistance to manipulation, corresponding to the Lance definition of spasticity (Lance, 1980).

However, Burne et al. (2005) stated that spasticity is due to enhanced baseline activity and therefore only observed under passive conditions. This implies that under active conditions, it is not hyperreflexia that is the problem, but modulation of afferent feedback as *a condition sine qua non* for proper motor function.

What is the substrate of this modulation and how does it work out in a functional way? Experiments identified the long latency reflex as the primary carrier of modulatory action (Pruszynski and Scott, 2012) as it was found to be dependent on instruction (Rothwell et al., 1980; Krutky et al., 2010), pharmacological agents like Tizanidine (Meskers et al., 2010), Transcranial Magnetic Stimulation (TMS; van Doornik et al., 2004; Pruszynski et al., 2011; Perenboom et al., 2015), task (Hallett et al., 1981) and scaled to task related urgency (Crevecoeur et al., 2013). According to optimal control theory, reflexes are adapted continuously and instantaneously based on manipulation of sensory information during voluntary movement (Pruszynski and Scott, 2012). Evidence for cortical involvement in the long latency reflex period is ample; whether modulation is located spinally of cortically is yet unknown (Figure 2, Perenboom et al., 2015).

## The Plant: Muscle and Passive Viscoelastic Structures

The musculoskeletal system in which muscle force is distributed can be regarded as the plant, the mechanical filter through which the neural controller comes to expression: high frequency modulations may be filtered by muscle activation and co-contraction when addressing effects of force production, e.g., in the hand or on the ground. Both position dependent elastic (spring) and velocity dependent viscous (damper) forces act on the masses of the limbs and environment. These forces are determined by non-contractile connective tissue and contractile muscle tissue, both in series and parallel to each other and dependent on state of activation (e.g., de Vlugt et al., 2010). The elastic properties of the connective tissue may be described by a logarithmic function; the elastic-like behavior of the muscle fibers behave according to the force-length characteristic originating from the sliding contractile filaments (Huxley and Simmons, 1971; Thelen, 2003). The velocity dependent properties are dominated by the specific force-velocity characteristic of the contractile tissue (Hill, 1938). However, for fast length changes these characteristics are not sufficient in describing muscle mechanics due to the phenomenon known as short range stiffness resulting in high stiffness over a short length range beyond which the muscle abruptly transits into a more viscous-like Behavior (Rack and Westbury, 1974; Campbell and Lakie, 1998; Cui et al., 2008; Van Eesbeek et al., 2010).

## Control and Plant Interaction

Control and plant interaction provide for different strategies for modulation of impedance, i.e., mechanical viscoelasticity from connective tissues, and/or mechanical viscoelasticity from continuous neural activation), and/or reflexive activations and/or co-contraction (Milner, 2002). Co-contraction provides instantaneous resistance, just as the connective tissues but is costly in terms of metabolic energy. Connective tissue in parallel to the muscle lacks control freedom, only substantially contributes in extreme joint angles. The serial tendon may store and release energy to amplify muscle power. Afferent feedback is energy efficient, but comes with a time delay that may threat postural stability. These strategic control possibilities require concerted action of supraspinal reflex control (with a time delay) and adjustment of internal models; the latter serves to improve movement properties (speed, precision, energy) from the use of *a priori* knowledge of the neuromechanical system (Wagner and Smith, 2008; Crevecoeur and Scott, 2014).

Although it becomes evident that upper motor neuron diseases related movement disorders are the result of a complex, environment- and task dependent interplay (Mirbagheri et al., 2004; van der Krogt et al., 2012) between primary neural and secondary biomechanical changes of muscles, tendons and connective tissue (Vattanasilp et al., 2000; Hof, 2001; Fridén and Lieber, 2003; Lieber et al., 2004; Gracies, 2005a,b; Dietz and Sinkjaer, 2007; Mirbagheri et al., 2009; Smith et al., 2011), clinical studies on the precise interaction, i.e., both temporal (dynamic stability analysis) and spatial (nonlinear dynamics), between controller and plant are scarce.

**Figure 3:**
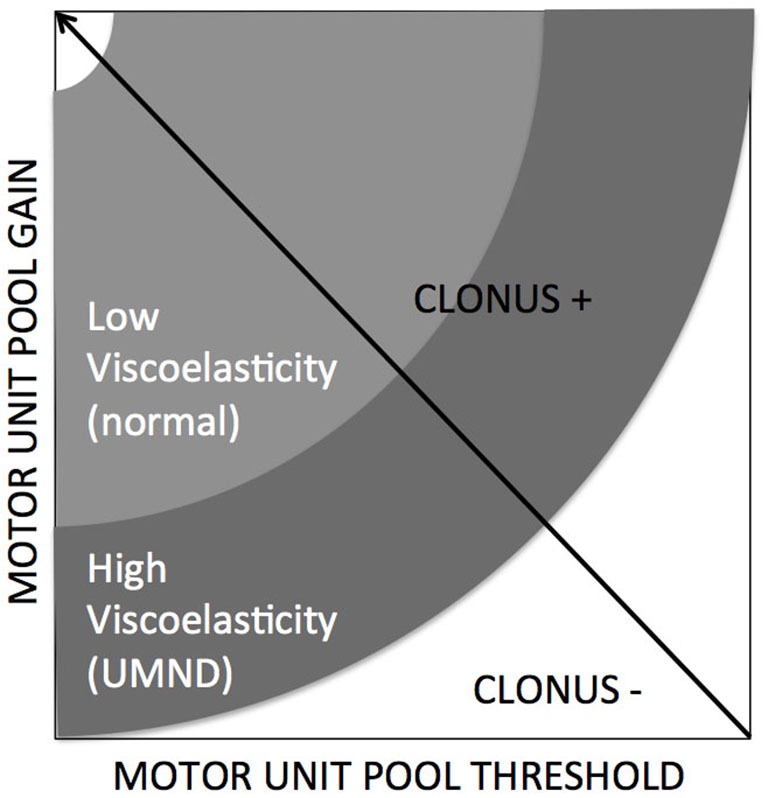
**Clonus is emerging when specific conditions are met, being combinations of neural factors (gain and threshold) and altered/increased tissue viscoelasticity (de Vlugt et al., 2012)**.

Kamper et al., 2001 addressed the potentiating effect of the mechanical part i.e., stiffening of intrafusal muscle on the controller. Increased size of motor units may stress the controller by violation of the Henneman’s size principle replacing a proportional with a strenuous “bang-bang” type of control (Hermes and LaSalle, 1969). From a control engineering point of view, decreased thresholds (Hidler and Rymer, 1999) or increased reflex gains result in mechanical instability of the controlled plant. A clear example of such an instability or oscillation is the phenomenon of clonus, a stereotypic, sustained, fast, repetitive and self-generated movement of mostly distal joints of patients with upper motor neuron diseases which is elicited by short force or torque perturbations by physicians or environment (e.g., floor contact). A simulation study showed that increased tissue viscoelasticity acting as an amplifier for increased reflex gain, by means of simulated changes in threshold and gain of the spinal motor unit pool is conditional for clonus (Figure 3, de Vlugt et al., 2012).

### System Identification and Parameter Estimation (SIPE) Techniques

Tight coupling between afferent sensory information, neural controller, efferent commands and motor properties makes it difficult quantify individual contributors by routine clinical examination (Lorentzen et al., 2010). Improper forces evoked by the neural system are either feedback related, i.e., movement velocity sensitive, feed-forward related i.e., improper voluntary control or by increased baseline muscle activity. Non-neural contributors are altered viscous (damper) and elastic (stiffness, spring) properties of contractile muscle and non-contractile tissue. Tissue properties may be modulated by neural activity. Neural activity is modulated by task instruction. Neural and non-neural components cannot be separated by task (instruction) alone, i.e., as under both passive vs. active conditions neural and non-neural contributors play a role, although their relative contributions will differ. Also, expression of neural and non-neural contributors is depending on limb manipulations in terms of acceleration, speed and position.

**Figure 4:**
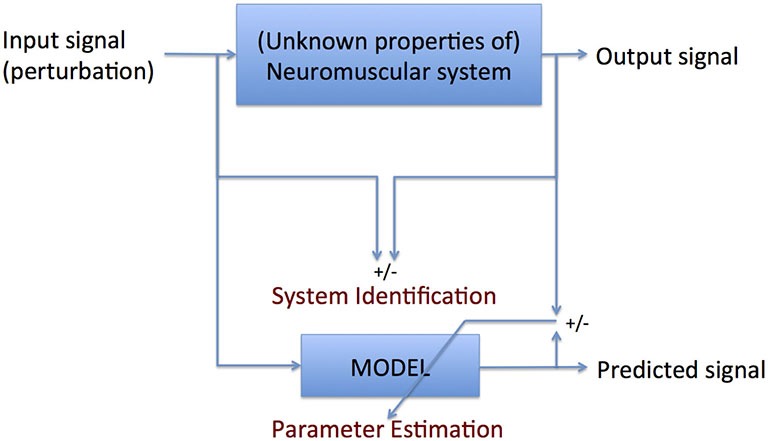
**Principle of system identification (SI): formal description of the comparison between input and output signal parameter estimation (PE): translation of the formal description into meaningful parameters**. SI requires no *a priori* knowledge of the system to be identified; PE does.

**Figure 5:**
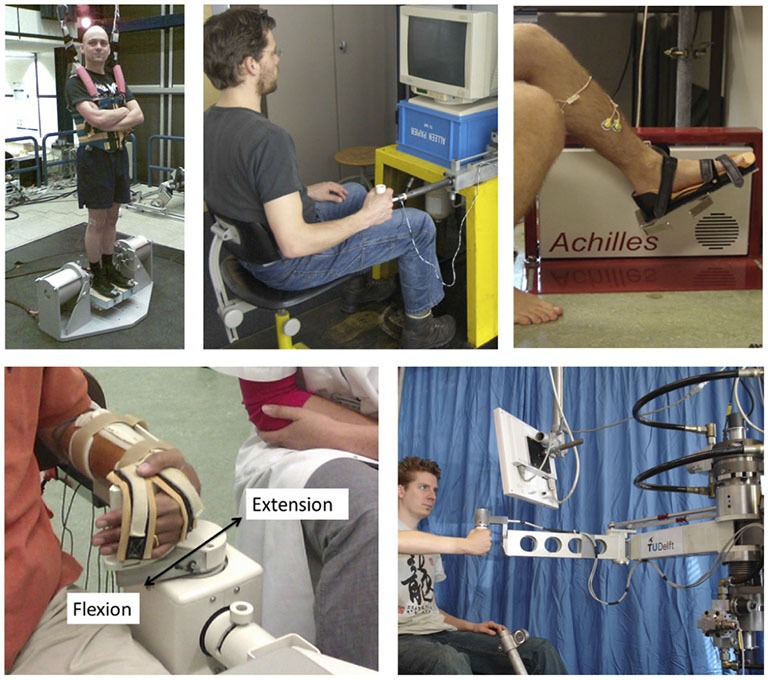
**Examples of robot manipulators used to apply position/angular or force/torque perturbations for SI purposes during stance (one Degree-of Freedom, DoF; rotational ankle perturbator; left upper corner), the ankle joint (one DoF rotational, upper right), the wrist joint (one DoF rotational, lower left) and the upper extremity (one DoF linear perturbator; upper middle and a two DoF; lower right corner)**.

**Figure 6:**
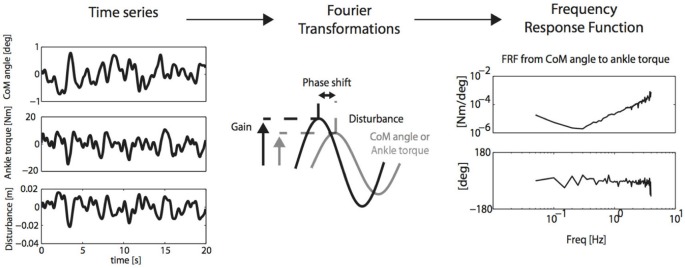
**SI and PE (SIPE): perturbation signals are related to CoM cangle and corrective ankle torque by Fourier transformation and calculation of the Frequency Response Function (FRF) of the neuro-muscular controller, describing gain and phase of aforementioned relations**. FRF’s may subsequently be translated into physiologically meaningful parameters by neuromuscular modeling (Engelhart et al., 2014).

Therefore, if the expression of the full interacting neuromuscular system is addressed, a SI approach is required. SI is the formal description of dynamical systems behavior derived from input-output relations (Figure 4, Kearney and Hunter, 1990; Kearney et al., 1997). Essential in (closed loop) SI is the application of precise and well-known external perturbations, applied by robot manipulators (Figure 5, Peterka, 2002; van der Helm et al., 2002; van der Kooij and van der Helm, 2005; Schouten et al., 2006; Palazzolo et al., 2007; Volpe et al., 2009; Balasubramanian et al., 2012). Black box identification approaches relate input perturbations to output signals, i.e., force, torque, position, angle, EMG to estimate integral system behavior. A *closed loop* system approach is a special form of SI that is required to prevent erroneous conclusions in case of cause and effect interrelations (van der Kooij et al., 2005; Westwick and Perreault, 2011; Campfens et al., 2013). This will be the case during functional tasks when the human controller is within the assessed loop and/or when the applied perturbations are part of the task. System responses may be directly, e.g., tissue properties and by constant neural activation or with a certain time delay i.e., reflexes. The differences between the response and the disturbance in means of amplitude (gain) and time delay can be displayed by a Frequency Response Function (FRF), which consists of two parts, a gain and a phase curve (Figure 6, e.g., Engelhart et al., 2014). For instance, during balance maintenance, a gain factor between a platform perturbation and resulting muscle activity, ankle torque or body sway is a valid way to express the overall performance of the balance control system. This gain factor is a measure of the resilience of the system (Engelhart et al., 2014). The phase curve discriminates between mass, spring damper characteristics of the system and identifies delayed neural controller related reflexive responses. Neuromechanical modeling can subsequently be fitted to FRF’s in a least squares sense to translate input-output behavior into physiologically meaningful parameters (van der Helm et al., 2002; de Vlugt et al., 2003; Schouten et al., 2008). Gray box approaches with pre-assumptions regarding underlying neurophysiology assist in further identification of individual components. Manipulation of the frequency content of the perturbation signal (van der Helm et al., 2002), virtual damping environment (de Vlugt et al., 2002; Meskers et al., 2009) or application of negative and positive force fields (Engelhart et al., Accepted) may specifically provoke or supress reflex activity. By manipulation of sensory channels the process of relative down-and unweighting of sensory information can be assessed (Pasma et al., 2012, 2014a,b) Assländer and Peterka, 2014; Multiple perturbations and multiple-input multiple-output (MIMO) System Identification and Parameter Estimation (SIPE; e.g., Perreault et al., 1999; Engelhart et al., 2014) are required to identify the contributions of individual limbs in a multi-link system and to assess different segmental control.

A particular problem of biological systems in general and neuromuscular system in particular is its non-linear (Kearney and Hunter, 1988; Palazzolo et al., 2007; Klomp et al., 2013) and time variant behavior. A clear example of non-linearity is the ability of humans to adapt to the environment and task demands. A solution is to linearize the system by reducing the work-space over which system properties are addressed (Mirbagheri et al., 2001; van der Helm et al., 2002; Schouten et al., 2008), i.e., very small changes in joint angle and muscle activation. The downside of this approach is that it is very hard to relate system behavior to functional tasks. Another example of non-linearity is related to the neurophysiology of the underlying structures, e.g., the unidirectional sensitivity of the muscle spindle. The neuromuscular system appears to be very sensitive to violation of linear assumptions (Klomp et al., 2014). Thus, non-linear models are required. These were successfully applied in open loop conditions (Van Eesbeek et al., 2010; de Vlugt et al., 2012; de Gooijer-van de Groep et al., 2013). Time variant behavior of stiffness and reflexes (Ludvig et al., 2011; van Eesbeek et al., 2013; Lee and Hogan, 2014) can be assessed using a cascade of linear models with time-varying parameters, so called linear parameter varying (LPV) identification (Verhaegen and Verdult, 2007).

### Clinical Application of SIPE

**Figure 7:**
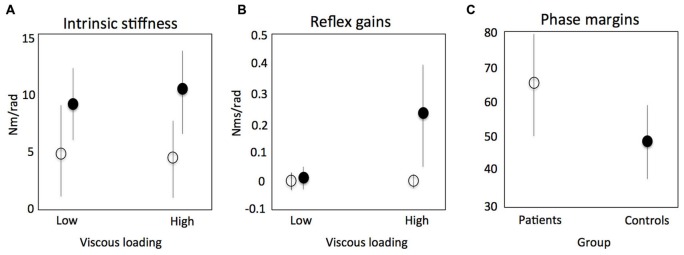
** Results of a study using system identification to identify intrinsic stiffness and reflex gains around the wrist in stroke patients**. Continuous random torque perturbations had to be resisted maximally. Intrinsic stiffness (elasticity) for controls (black circle) and patients (open circle) respectively **(A)**; reflex gains (kv) as a function of increasing viscous load **(B)**; phase margins as a measure of the mechanical (in) stability of the addressed control loop, i.e., the tendency to oscillate, estimated by calculating the phase shift (phase margin) needed to reach instability of the total system of manipulator and subject **(C)**. Meskers et al. (2009).

There are potentially three clinically relevant applications of SIPE: (1) understanding of pathophysiological mechanisms that determine the relation between initial neural damage and its functional consequences; (2) assessment to select proper therapy; and (3) biomarkers for prediction of (functional) outcome and early predictors of therapy success. During active task conditions, evidence was found for impaired reflex modulation in the upper limb in stroke patients (Meskers et al., 2009, Figures 7B). This in is concordance with earlier findings using neurophysiological techniques (Mazzaro et al., 2007; Trumbower et al., 2013) and SI under passive conditions (Mirbagheri et al., 2001). In one particular experiment, patients were asked to maximally resist random force perturbations applied to the handle of a one degree-of-freedom (DOF) haptic wrist perturbator (Schouten et al., 2006). Subjects were visually informed on the position of the handle for motivation purposes. Linear SI and neuromuscular modeling fitting the perturbation signal to the resulting angular wrist rotations were used to identify main characteristics of the reflex loop, i.e., velocity dependent reflex gain, time delay and intrinsic stiffness and viscosity. In this study the stiffness component was a combination of tissue properties modulated by non-velocity dependent neural activation. Stroke patients therefore showed lower stiffness compared to healthy controls as a reflection of the paresis, e.g., the decreased capacity for active torque production (Figure 7A, Kamper et al., 2006). Phase margins were calculated as a measure of the mechanical (in) stability of the addressed control loop, i.e., the tendency to oscillate, estimated by calculating the phase shift (phase margin) needed to reach instability of the total system of manipulator and subject. Increased stability of the reflex loop in stroke patients was found (Figure 7C). This adds to the evidence that functional improvements after stroke are primarily the result of compensation strategies with the unaffected limb (de Haart et al., 2004; Buurke et al., 2008; van Kordelaar et al., 2012, 2013). Also, evidence was found that elderly reduce postural responses to perturbations less compared to young subjects in case of increasing external force fields (Engelhart et al., Accepted).

Separation of the contributing factors to joint stiffness, i.e., intrinsic stiffness and reflex stiffness is important when choosing the right strategy to reduce increased joint stiffness in stroke (Mirbagheri et al., 2001; de Vlugt et al., 2010) and CP (de Gooijer-van de Groep et al., 2013; Sloot et al., 2015). Measurements in CP and controls showed on average a 5.7 times larger (*p* = 0.002) reflex torque and a 2.1 times larger tissue stiffness (*p* = 0.018) compared to controls (de Gooijer-van de Groep et al., 2013). There was a trend of increased reflex and baseline activity in the clinically high graded spastic group and decreased reflex and background activity after spasticity treatment with botulinum toxin (Sloot et al., 2015). In stroke patients, triceps surae tissue stiffness was increased about three times and on average a five times increase in reflex torque was found. Differences in “type” of spasticity, i.e., ratio between tissue stiffness and reflex torque, between CP and stroke, may add to the understanding of this phenomenon; high variability in patients which scales with clinical phenotype may be the basis for a better selection of patients for treatment: the patients with relatively high reflex torque with reflex blocking agents and the patients with a relatively high tissue stiffness with casting, splinting and orthopedic surgery.

A recent study in healthy elderly, elderly with cataract, polyneuropathy and balance disorders using bilateral angular perturbations of a leg support surface showed specific responses of body sway and ankle torque (Pasma et al., 2014b). These responses allowed for calculation of sensory weighting i.e., the relative down- or up regulation of information of one sensory channel over the other. It appeared that proprioceptive information is weighted more with age, cataract and with impaired balance. In patients with polyneuropathy and with impaired balance proprioceptive information reweighting increased with the amplitude of disturbances. These results show the opportunity to detect the underlying cause of impaired balance in elderly using SI techniques and to apply target interventions to improve standing balance.

Neuromechanical parameters around the wrist respectively the elbow were shown to be predictors of functional outcome of arm-hand function after stroke as assessed by the Action Research Arm Test (ARAT; van der Krogt et al., 2015) and Fugl Meyer Assessment (FMA; Mirbagheri et al., 2012). These are the first steps to fully work down joint and limb function in its basic neuromechanical parameters, preferably under both passive and active conditions. Application of SIPE and addressing neuromechancial parameters longitudinally after events like stroke may substantially add to precision diagnostics, which will allow for application of the right therapy at the right time. Proper knowledge of the dynamics of neuromechanical properties in relation to functional outcome may facilitate assessment of the effects of novel, high tech and costly new treatment paradigms like robot training that currently do seem not to surpass that of conventional training (Kwakkel and Meskers, 2014). Identification of primary neurological repair vs. compensation is crucial. In stroke patients, the contribution of the paretic leg to resist external perturbations was found to be significantly smaller than the contribution of the paretic leg to weight bearing (van Asseldonk et al., 2006). These approaches may yield biomarkers to optimise therapy, which may require combinations of assessment- and training robots (Balasubramanian et al., 2012). Assessment during functional, active tasks is required when aiming for functional improvement. This requires closed loop approaches or SI using perturbations, which do not interfere with the task (Burdet et al., 2000). Non-linear and time variant SI is required as linear approaches are easily violated (Klomp et al., 2014). Combining peripheral perturbations with neurophysiological measurements like Electro Encephalography (EEG) yields properties of the sensor-controller-motor loop in more detail. Assessing cortical responses to fast muscle stretches yields stretch Evoked Potentials (strEP) that may serve as a measure of cortical sensorimotor activation in response to proprioceptive input (Campfens et al., 2015a). Afferent sensory pathway information transfer and processing can be assessed by calculation of the coherence between cortical activity and a peripheral position perturbation (position-cortical coherence, PCC; Campfens et al., 2015b). Aforementioned measures are disturbed in stroke patients and may be used to detect integrity of afferent and efferent pathways separately and propagation of signals over the cortex (Campfens et al., 2015a,b). High density EEG may further reveal cortical involvement and its location in motor tasks (Yao and Dewald, 2005a; Yao et al., 2005b).

Besides the short-term interactions within instantaneous movement control, the long-term interaction between the controller and the plant is still underexposed. The interaction between the quality of the contractile tissues and cognition in aging, the impact of acute neural deprivation after stroke on contractile tissue properties and the unbalanced growth of skeletal and contractile tissues in CP may result in long term interrelated changes that are currently described but of which the mechanisms are yet not understood. This requires long term follow-up of patients with upper motor neuron disease and the process of ageing.

### Future Work

Assessment and understanding of proper modulation of joint impedance to the task at hand as cornerstone of improving level of activity in patients sets the future direction. Questions to be addressed, comprise: (1) What are underlying pathophysiological mechanisms of impaired modulation and can these be worked down into the basic neural and mechanical components; (2) Does identification and quantification of these components translate into specific targets for intervention to improve function in patients with neurological diseases? and (3) Can these components serve as high-resolution biomarkers for prediction of (functional) outcome and early predictors of therapy success? In order to meet clinical demands, supraspinal motor control needs to be addressed in conjunction with sensor and motor characteristics taking environment and task into account. Only in this way we are able to understand the complex system interactions of primary deficits and compensations that underlie motor disorders in central neurological diseases. Neuromechanics play a key role. SI is a preferred tool for assessment. We are entering an exciting field of research, which may prove a key to fundamentally understand mobility disorders. The clinical urgency is clear, both qualitatively and quantitatively.

## Conclusion

There is an urgent clinical need for assessment, identification and targeted intervention for disability inducing posture and movement disorders in neurological disease and ageing. What we fundamentally have to address is the underlying inappropriate interaction with the physical environment with inadequate neuromuscular force and impedance control of the patient. This requires assessment of both the neural controller component and the mechanical, “motor” component and most importantly their interaction. Promising tools are (closed loop) SI techniques to address neuromechanics in response to well defined environmental tasks and conditions. Clinical application is yet scarce, yet demanded.

## Author Contributions

CGMM wrote a draft of the manuscript and performed the subsequent and final edit. JHDG, EDV and ACS wrote parts of the manuscript and commented on versions of the manuscript.

## Conflict of Interest Statement

The authors declare that the research was conducted in the absence of any commercial or financial relationships that could be construed as a potential conflict of interest.
